# Development of mobile health-based interventions to promote physical activity in patients with head and neck cancer: a qualitative study

**DOI:** 10.3389/fpubh.2023.1260804

**Published:** 2023-11-22

**Authors:** Yan Ning, Zhen Dong, Zehuan Jia, Wenting Zhao, Yongxia Ding, Qian Wang, Ruifang Zhu, Shifan Han

**Affiliations:** ^1^School of Nursing, Shanxi Medical University, Taiyuan, China; ^2^Department of Otolaryngology Head and Neck Surgery, First Hospital of Shanxi Medical University, Taiyuan, China; ^3^Shanxi Key Laboratory of Otolaryngology, Head and Neck Cancer, Taiyuan, China; ^4^Editorial Department, First Hospital of Shanxi Medical University, Taiyuan, China

**Keywords:** head and neck cancer, mHealth, physical activity, UTAUT, qualitative study

## Abstract

**Background:**

Despite the well-grounded benefits of physical activity (PA), poor compliance with the PA guidelines has been reported among head and neck cancer (HNC) patients. Mobile health (mHealth)-based interventions can help cancer survivors increase their PA levels and increase the reach or efficiency of rehabilitation services. However, there is limited knowledge about the needs and perceptions of HNC patients regarding these interventions. This study explored the perceptions and needs of HNC patients regarding mHealth-based PA programs before developing such interventions to ensure their improved effectiveness.

**Study design:**

A constructivist qualitative study.

**Methods:**

We purposively selected 17 adult HNC patients aged 40–80 years to determine their needs and perceptions of future mHealth-based PA programs. Semi-structured face-to-face interviews were conducted, and the data were analyzed via thematic analysis. The report followed the Consolidated Criteria for Qualitative Research Reports guidelines.

**Results:**

Four themes were analyzed from the interview transcripts regarding the needs and perceptions of mHealth-based PA programs: (1) functionality needs; (2) system usage requirements; (3) social support; and (4) perceived barriers and facilitators. HNC patients expect highly customized and specialized mHealth interventions that consider individual factors, address their concerns about security, network, and cost, and prefer improved comfort. Moreover, they expect to receive support from their healthcare providers, families, and peers.

**Conclusion:**

The study provides pragmatic ready-to-use recommendations to design interventions for inactive HNC patients to achieve the recommended PA levels. Future mHealth interventions should be tailored according to the needs of the HNC patients by utilizing perceived facilitators and removing perceived barriers to help them engage in PA actively.

## 1 Introduction

Head and neck cancer (HNC) is the sixth most frequent cancer type worldwide (1). In China, approximately 115,000 new HNC cases were diagnosed in 2014, including 84,000 men and 31,000 women. The crude incidence rate was 8.32/100,000 (2). With recent advances in cancer treatment and HNC management, the 5-year survival rate of HNC is estimated as 40–50% (3, 4). Financial toxicity is a significant issue for some HNC patients, which is associated with a reduced health-related quality of life (5). Additionally, patients with HNC are at significant risk of cardiovascular events; hence, cardiovascular risk mitigation is essential to survivorship care (6).

Physical activity (PA) is considered an effective non-pharmacological treatment to improve the health status of HNC patients. Higher levels of PA were associated with a 7% reduction in the overall cancer risk (hazard ratio [HR] = 0.93, confidence interval [CI]: 0.90–0.95), with a moderate negative association (10–20% risk reduction) observed for HNC (HR = 0.85, CI: 0.78–0.93) (7). PA can be an effective intervention that complements the treatment and prevents treatment-related cardiovascular disease (8). Moreover, PA during or after treatment in HNC patients significantly improves the functional capacity, prevents the worsening of fatigue, and improves the quality of life (9). PA interventions in cancer survivors are cost-effective, with cost-effectiveness better than US$ 101,195 per quality-adjusted life years gained (10).

Despite the well-documented benefits of PA, adherence of HNC patients to the recommended PA guidelines is poor (11). Objective barriers experienced by HNC patients in performing PA include time pressure, distance to program, and lack of facilities or space. Low self-efficacy and lack of knowledge also prevent patients from participating in PA (12). Additionally, the cultural context is an important factor affecting patients’ PA preferences; for example, patients’ perceptions of PA in traditional Chinese culture may act as an implicit barrier to exercise (13). Moreover, with the coronavirus disease 2019 pandemic and regulations enacted to reduce its spread (e.g., home isolation, gym and recreation center closures, and social distancing) (14, 15), the preexisting pandemic of physical inactivity has become more serious. Therefore, using expanded telehealth to provide non-traditional care delivery models has been encouraged (16).

The use of mobile health (mHealth) as a core public health tool has increased dramatically in healthcare, because it changes the environment for patient care and the delivery of healthcare, addressing the geographic and logistical barriers of traditional programs and improving the efficiency of rehabilitation services (17, 18). The mHealth-based PA interventions may be safe and feasible to help HNC patients improve their PA levels and compatible with concurrent chemotherapy or radiotherapy treatment (19, 20), however, compliance of HNC patients with mHealth programs for PA has been reported as 50–70% (21). The willingness of patients to use mHealth programs will be stronger if they believe that it is useful and easy to use (22, 23), and they are often subject to the social influence around them when making mHealth decisions (24). However, there is limited knowledge about the needs and perceptions of HNC patients regarding these interventions. Therefore, it is needed to explore the patients’ needs and perceptions with respect to four core factors influencing user acceptance (performance expectations, effort expectations, social influence, and convenience) based on the Unified Theory of Technology Acceptance and Use (UTAUT) (25). The UTAUT explains more than 70% of the variance in behavioral intentions and outperforms eight existing models.

In this study, we aim to explore the needs and perceptions of HNC patients regarding mHealth-based PA programs before developing such interventions, which was conducted to inform a pilot feasibility study to provide support for PA to HNC patients. The findings of this study could provide the limited evidence needed to promote digital adherence techniques and public health education.

## 2 Methods

### 2.1 Study design

A constructivist qualitative methodology (26) was used, and the data were analyzed via thematic analysis (27). A constructivism-based qualitative research approach was chosen because it proposed that the results of the study are a product of the participants’ experiences and understanding and their interaction with the researcher sharing the phenomenon. Moreover, it facilitated determining the patients’ needs and perceptions regarding the emerging mHealth interventions in the context of Chinese culture. This study followed the Consolidated Criteria for Reporting Qualitative Research guidelines (28) for reporting (Supplementary File S1).

### 2.2 Study setting and participants

This study was conducted at the Otolaryngology Department (Department of Otorhinolaryngology–Head and Neck Surgery) and the Oncology Department of the First Hospital of Shanxi Medical University in Taiyuan. Participants were recruited in person by the second author from June 20 to November 15, 2022. Purposive sampling produced a sample of 17 patients with HNC. This study aimed to inform the future development of a PA intervention that utilizes mHealth; therefore, we intended to recruit HNC survivors who were hospitalized patients or were hospitalized after discharge and would benefit the most from these interventions (i.e., insufficiently active patients) (29). The inclusion criteria were as follows: (1) age > 18 years; (2) diagnosed with HNC and receiving adjuvant therapy or having completed adjuvant therapy (i.e., chemotherapy and/or radiation therapy); (3) insufficiently active (i.e., engaging in <150 min of moderate-intensity PA per week in the past month based on self-report); and (4) ability to walk without pain or discomfort. Patients were excluded if they had self-reported health issues that limit PA or if they were unable to speak.

We used purposive sampling methods to approach participants for diversity in the sample characteristics. For instance, we considered the disease stage, treatment method (surgery or radiotherapy), area of residence (urban or rural), and different economic levels as relevant characteristics for the HNC patients.

### 2.3 Data collection

Data were collected by conducting face-to-face semi-structured interviews and audio-recorded by YN, who was a nurse trained in gathering qualitative data, study objectives, managing dialog, and interaction with participants of various dispositions during their nursing PhD course. Similarly, fundamental training was conducted for transcription, coding, and analysis. The interviews were conducted in a private conference room, with no one else present during the interview. All interviews were conducted at a time best suited for the participants. Before beginning each interview, the researchers read out the participant explanation statement to the patients, following which the participants provided written informed consent. Researchers recorded field notes at the end of each interview. The mean interview time was 35 min. Participant interviews continued until the data were saturated. All interviews were recorded with prior permission obtained from the participants.

### 2.4 Interview guides

A hypothetical scenario was used to describe the general content of the mHealth-based PA program, as none of the participants had prior experience with it. This hypothetical scenario was presented verbally once at the beginning and repeated on the participant’s demand during the interview if they needed to recall it. The mHealth-based PA program comprised three main components as follows: a patient-facing component consisting of a smartphone application to record the patient’s daily PA and provide PA-related health education; a secure Health Insurance Portability and Accountability Act-compatible cloud server storing encrypted videos; and a computer-based login system to monitor the patient’s daily PA and confirm compliance.

The interview guide was guided by the UTAUT (Table 1). It included open-ended questions about the patient’s needs regarding performance and effort expectations when using the mHealth-based PA intervention program in the future, perceptions of social influence, and perceived barriers and facilitators. The interviews were conducted in Mandarin Chinese, and the quotes have been translated into English. Two interviews that were initially conducted as pilot interviews were included in the analysis for their content, as they covered valuable information relevant to the study objectives. After completing the pilot interviews, minor changes were made to the interview guide, such as starting each interview by focusing on the smartphone and mHealth use in daily life to allow patients to understand the topic of the study better and be more open-ended.

**Table 1:** Interview guide.

Construct	Item	Description
Performance expectancy	PE1	Would you need an mHealth system to help you with your PA?
	PE2	What customization features would you like to see in your mHealth-based PA program?
	PE3	Is there any feature or function you would consider very important for inclusion in a good mHealth system that would stimulate your interest to use it regularly?
Effort expectancy	EE1	What makes/made the information in the mHealth systems clear and understandable?
	EE2	What makes/made the mHealth systems easy to operate?
Social influence	SI1	Is there anyone within your social circle who supports you in using mHealth systems?
	SI2	Is there anyone important to you who thinks that you should use mHealth systems?
Facilitating conditions	FC1	What do you find challenging about using mHealth systems for PA management?
	FC2	What challenges or facilitators do you find in terms of the necessary resources needed to use the mHealth system?
	FC3	What challenges or facilitators do you find in terms of the necessary knowledge needed to use the mHealth system?
	FC4	Is there a specific person (or group) available to help you when you encounter difficulties with the mHealth system?

HCPs, healthcare professionals; mHealth, mobile health; PA, physical activity.

### 2.5 Data analysis

Immediately after each interview, the researcher (YN) transcribed the recording verbatim and compared it with the original recording to ensure accuracy. Two researchers (YN and ZH J) performed a reflexive thematic analysis following the six phases described by Braun and Clarke (30) to analyze the data. As thematic analysis is very flexible and facilitates distinguishing, identifying, and interpreting themes, we considered it appropriate for use with interpretive descriptive methodology. Qualitative analysis was managed using NVivo 12. The verbatim transcripts were read several times to develop a broad understanding of the participants’ responses. Following this, the words, phrases, and statements were coded. The codes and phrases were clustered in themes using reflexive thematic analysis, code refining, and combining into subthemes. The subthemes were refined and collated to develop themes, based on relevance, content, and meanings.

### 2.6 Ethical considerations

The study was conducted according to the Dutch law and the Declaration of Helsinki. Approval for the study was granted by the Ethics Committee of First Hospital of Shanxi Medical University (2022/K193). All participants provided written informed consent before the interviews. Our data, including the interview transcripts, did not include the participants’ names or other identifying information to protect and maintain their confidentiality. All of our findings are presented anonymously, with only the research team having access.

### 2.7 Rigor

Several strategies were used to ensure credibility, transferability, confirmability, and dependability (31). Meetings with all researchers were held at each level of coding to discuss the coding and grouping of codes into themes to enhance interpretative rigor. The literature review, pilot interviews, and discussions among researchers facilitated the development of interview guidelines. The second author had prolonged contact with the participants, leading to reasonably long interviews. The researcher’s training in qualitative research ensured the credibility of the research instrument. Two researchers conducted the analysis independently, while a third validated the themes and structure (researcher triangulation).

## 3 Results

This study included 17 participants, and their characteristics are presented in Table 2. The age range of the participants was 40–80 years, and most of them were men (14/17). Oral or nasopharyngeal cancer was diagnosed in 12/17 patients, and 13/17 patients had Stage III–IV cancer. Most of the patients (13/17) had received radiotherapy or chemotherapy.

**Table 2:** Characteristics of the participants.

Variables	*n* (%)
Sex	
Male	14 (82.4)
Female	3 (17.6)
Age (years)	
40–50	7 (41.2)
51–60	4 (23.5)
61–70	4 (23.5)
≥71	2 (11.8)
Education level	
Lower (primary education or less/lower secondary)	12 (70.6)
Intermediate (upper secondary/post-secondary non-tertiary)	1 (5.9)
Tertiary (short-cycle tertiary/bachelor/master/doctoral)	4 (23.5)
Marital status	
Married	17 (100.0)
Divorced	0 (0)
Widowed	0 (0)
Place of residence	
City	8 (47.1)
Countryside	9 (52.9)
Cancer site	
Larynx	3 (17.6)
Oral cavity	6 (35.3)
Nasopharynx	6 (35.3)
Others	2 (11.8)
Cancer stage at diagnosis	
Stage I	1 (5.9)
Stage II	3 (17.6)
Stage III	5 (29.4)
Stage IV	8 (47.1)
Treatment type	
Surgery only	4 (23.5)
Chemotherapy only	2 (11.8)
Radiotherapy only	3 (17.6)
Chemotherapy + radiotherapy	2 (11.8)
Radiotherapy + surgery	6 (35.3)

**Figure 1:**
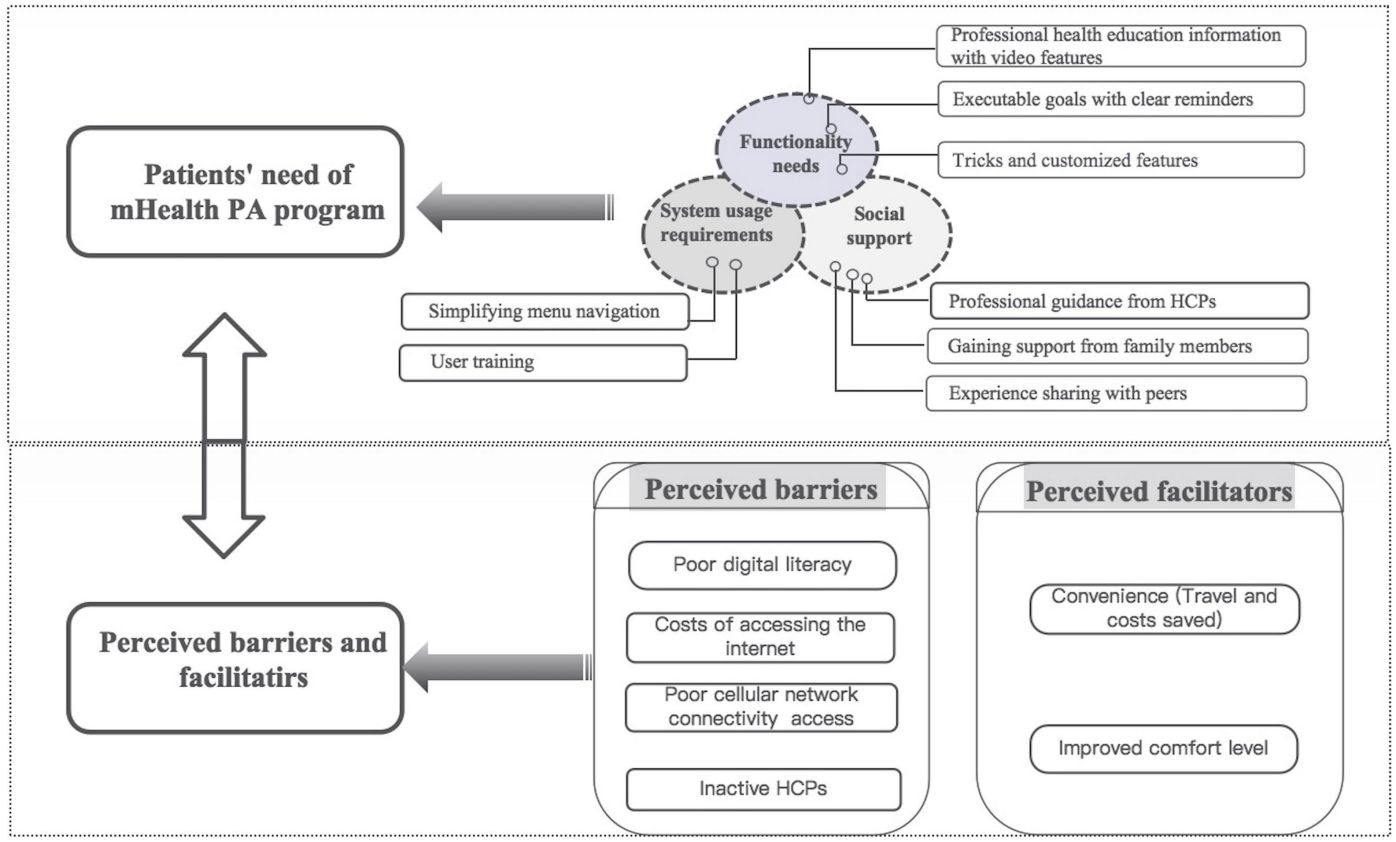
Themes and subthemes of the needs and perceptions of HNC patients regarding mHealth-based PA programs.

The following four themes were analyzed from the interview transcripts: (1) functionality needs; (2) system usage requirements; (3) social support; and (4) perceived barriers and facilitators. Figure 1 shows the themes and subthemes, and these are discussed in detail with illustrative quotations of participants below.

### 3.1 Functionality needs for the mHealth PA program

When considering how to improve the perceived usefulness of mHealth-based PA programs for HNC patients, participants expressed their needs for functionality, which included professional health education information with video features, executable goals with clear reminders, and tricks and customized features.

#### 3.1.1 Professional health education information with video features

Many participants agreed that although they knew the benefits of exercise for the general population, they wanted information that was more specific about the safety and benefits of exercising while undergoing treatment or after treatment. Specific information on how PA can help manage or mitigate side effects and how to perform it safely would be helpful and motivating. Furthermore, participants wanted to access user-friendly video demonstrations of exercises to help “implement different types of exercises” and to “push them to be better at exercise.”

*“Recovery from illness is a long process. I want to know information about when I can exercise, what exercise I should be doing, and how to do it safely, and that’s the most valuable feature of this app and distinguishes it from other commercially available exercise apps.” (Participant #10, young male).*

#### 3.1.2 Executable goals with clear reminders

Some patients felt that achievable goals should be set for them in the application that can be adjusted over time along with clear reminders. Participants undergoing chemotherapy indicated that they might suffer chemotherapy side effects, such as nausea, fatigue, and anxiety, which vary within and between treatment cycles among the patients. Fatigue often causes patients to feel limited in energy, and most of their energy is prioritized for family or daily activities; hence, it is important to consider this when designing realistic PA goals. They hoped that the interventions would allow them to “accomplish their PA goals” by providing guided structured plans that “suggest what you should be doing at each stage.”

*“Because I feel tired during and after radiotherapy, I will gradually get better until the next radiotherapy treatment. So I would like to be flexible with the exercise goals set in the app.” (Participant #11, young male).*

*“…and it would be helpful if there was an app that could remind me in the morning that it’s time to exercise, or sometimes if I ignore the reminder, it would remind me again in the noon or afternoon, three times a day.” (Participant #10, young male).*

#### 3.1.3 Tricks and customized features

Participants expected that their PA program be customized based on individual factors, including preferences, PA history, treatment characteristics, and symptoms. They also mentioned several strategies for maintaining their PA routines. Participants felt that maintaining a log during PA could be beneficial to the individual as it would “record and track (their) movements,” and they could “visualize their progress” and “measure how far they are from their goals.” They also reflected on how sharing a journal can not only help “monitor themselves at home” but also gain acknowledgment from their HCPs.

*“I used to play ping pong and enjoyed it. Although I am undergoing radiation therapy, I hope that my exercise plan will take into account my preferences and previous intensity.” (Participant #13, young male).*

*“Keep track and record what you have done so you can see and say, ‘I’ve done it, I’ve accomplished my goal for the day’…I feel very accomplished with PA, and it makes me feel confident about myself.” (Participant #11, young male).*

### 3.2 System usage requirements for the mHealth PA program

#### 3.2.1 Simplifying menu navigation

Regarding making the system easy to use, patients mainly mentioned simplifying menu navigation and reducing the user’s time investment.

*“The first look is the clickable buttons that allow us to go directly to the useful operating interface.” (Participant #10, young male).*

The application’s ability to save and display previously recorded data will also save time.

*“The apps can save all your previous records, so you can eliminate repetitive tasks, such as your account information and system preferences.” (Participant #17, young male).*

#### 3.2.2 User training

Before piloting these mHealth systems, patients expect formal user training to increase usability and uptake. One patient asked, “How do you use what you do not understand? Is there anyone to teach us?” Another emphasized, “Some systems are very difficult to navigate.”

*“I am not proficient in software and hardware usage. I wish someone had taken the time to educate us on how to use these systems, instead of assuming we know.” (Participant #12, older male).*

### 3.3 Social support for the mHealth PA program

Patients expressed the need for social support from healthcare providers (HCPs), family members, and peers. The engagement of HCPs to interact with patients and answer questions was considered important. Additionally, educating family members about the safety and efficacy of PA could help patients gain better support. Peer interaction and experience sharing can create an atmosphere of mutual support for PA.

#### 3.3.1 Professional guidance from HCPs

Participants felt that they would have a sense of safety and security if HCPs engaged in interactions and provided professional guidance to solve problems at any time. Participants also expressed the value of having a “WeChat-like group that includes doctors and patients,” where they can ask questions and get “instant responses” from doctors, which could serve as a reference for others with the same problem.

*“Being able to receive professional guidance from HCPs during my exercise was helpful and really appealed to me to use the app.” (Participant #9, older male).*

*“I was more concerned about the interaction with the doctors, and their support would give me a sense of confidence.” (Participant #11, young male).*

#### 3.3.2 Gaining support from family members

Participants stressed the need to be supported by family members when making treatment decisions and for PA management. For many patients, especially older patients or those with limited digital skills, their family members often encouraged them to rest and reduce activity. This was possibly due to filial and piety considerations in traditional Chinese culture or for safety and avoiding infection risk. Patients felt that educating family members on the safety and efficacy of PA after illness and transforming their traditional attitudes could help them gain better support.

*“Since I’ve been sick, my family has encouraged me to sit or lie down and rest, except for basic daily activities, because they feel that activity drains my energy, and they also feel that it’s unfilial for me to do housework when I’m sick.” (Participant #16, older male).*

*“I was quite fond of exercising but since I’ve been sick, my family has told me to exercise as little as possible. They think exercise interferes with energy recovery and feel it’s unsafe. I need their support which is important to me.” (Participant #10, young male).*

#### 3.3.3 Experience sharing with peers

Participants expressed the need for interaction with peers within the application. They explained the importance of support from peers with similar illnesses and treatment experiences who were able to share their PA experiences within the application, creating a sense of group belonging, empathy, and a social PA environment.

*“A forum for sharing and communicating with fellow patients is needed. In a sense, it’s like you belong to a group. I can share my feelings and have people who understand me, and I’m not alone.” (Participant #1, young female).*

*“When you are on your own, it’s harder because you encounter problems, and you might want to give up. But when you do it with other people, it’s much easier to have them as a reference point, or questions that can be answered in a timely manner.” (Participant #9, older male).*

### 3.4 Perceived barriers and facilitators

#### 3.4.1 Perceived barriers

##### 3.4.1.1 Poor digital literacy

Patients were digitally illiterate or had poor digital literacy with no appreciation for the relationship between mHealth and PA. For instance, some older patients may find telemedicine a challenge, as the task requires the ability to operate a cell phone and respond appropriately to their PA measurements.

*“I do not normally use my phone for everyday operations, such as surfing the web or shopping online. I only use it for phone calls and voice chatting in WeChat, because it is very difficult for me to learn those operations, and we older people are not able to keep up with the changes of the times.” (Participant #3, older male).*

##### 3.4.1.2 Costs of accessing the internet

Participants expressed concerns regarding the costs related to using mHealth. Most patients indicated that although they could connect to wireless internet at home, the recurrent costs of internet access outdoors were the main barrier. Some low-income patients expressed concerns that the internet they purchased would not be enough for their use outdoors and extra purchases would be required.

*“There is a situation where I have to connect to a cellular network for recording during my outdoor exercise. At this point, the cellular network data I purchased can easily be used up, and the excess will bring extra expenses.”* (*Participant #17, young* male).

##### 3.4.1.3 Poor cellular network connectivity and internet access

Some patients were concerned about inconsistent cell phone network coverage in certain areas. They indicated that an unstable network could interfere with the recording of the PA and thus affect their judgment of whether they had completed their daily PA goal.

*“There are some areas with poor network signal, slow internet speed, and at times, there is no network at all…If there is no network or internet, my exercise records will be impaired and how do I know if my PA goal is accomplished?” (Participant #1, young female).*

##### 3.4.1.4 Inactive HCPs

Most patients perceive that HCPs are unresponsive and poorly motivated to their needs due to their work overload. With this perception, the system, even if fully functional, would not work due to the need for direct interaction with the HCPs.

*“…It is even worse when asked about the topic of PA, as many HCPs see it as extra work.” (Participant #4, young male).*

*“It’s very frustrating to go to a hospital and find doctors and nurses very busy. What’s more, how about a platform that relies heavily on the availability of healthcare providers to function?” (Participant #6, young female).*

#### 3.4.2 Perceived facilitators

##### 3.4.2.1 Convenience (travel and costs saved)

Patients who are geographically isolated and have poor access to transportation, such as those living in rural areas, often consider mHealth-based systems beneficial. Moreover, the costs incurred due to trips to the hospital, or even the need to stay in a hotel, can be saved.

*“Parking at the hospital is inconvenient due to traffic jams. It would be a great relief if I could let my doctor know about my condition and adjust my PA* via *mobile phone so I do not always have to take (my son’s) car to the hospital, and I do not suffer from congestion and waste time and do not have to pay parking fees or toll fees.” (Participant #3, older male).*

##### 3.4.2.2 Improved comfort level

Patients with HNC are prone to facial disfigurement during the treatment process, which could make them feel uncomfortable and embarrassed in public. These feelings can make them reluctant to exercise; hence, mHealth-based PA can solve this problem. Additionally, patients felt less nervous in a remote situation than in a face-to-face conversation in the hospital.

*“The way I look now (the change in body image), I do not want to be in a crowded place or even be seen by a doctor. If I know I need to exercise, I want to do it in a quiet place. Communicating with the doctor remotely* via *mobile phone without having to meet face-to-face would avoid embarrassment.” (Participant #2, young male).*

## 4 Discussion

This study examined the needs and perceptions of HNC patients for developing mHealth-based PA intervention programs. Data analysis identified functionality needs, system usage requirements, social support, and perceived barriers and facilitators for the widespread use of mHealth-based PA programs, providing the first step in designing mHealth-based PA interventions for inactive HNC patients to achieve recommended PA levels. Overall, HNC patients expect highly customized and specialized mHealth interventions that consider individual factors, address their concerns about security, network, and cost, and prefer improved comfort. Moreover, they expect to receive support from their HCPs, families, and peers.

As for the functionality needs, patients particularly emphasized the importance of health education. Targeted educational and informational content for specific groups in mHealth interventions may increase disease awareness, bridge knowledge gaps, and encourage healthy behaviors (32), which is consistent with the findings of our study. Furthermore, our research found that especially in older or poorly literate patients, video-based education has gained popularity because the vividness of the videos provides clear learning content and motivates patients to obtain information on their own, as reported in other areas of global healthcare (33). The mHealth strategies comprising educational videos and video consultations could reduce the use of unplanned medical services and aid in rapid patient-reported recovery (34).

Our findings suggest that patients undergoing treatment or have completed treatment may want HCP engagement and access to tailored programs to ensure the safety and effectiveness of PA. Moreover, patients undergoing treatment expressed the need for additional programs in case of acute treatment-related side effects, such as fatigue and nausea. However, there is currently a serious imbalance between the number of patients and doctors and nurses in China (35), restricting effective communication between HCPs and patients. Patients felt that HCPs sometimes ignored their needs and delayed providing services to them because they were too busy at work. Many mHealth technologies fail to be adopted because HCPs are poorly equipped with digital technology and deliberately avoid the system because they perceive it to be time-consuming and want to maintain the status quo (36). Therefore, increasing the awareness of the clinicians and nurses regarding the tools available and engaging in digital training to develop the necessary skills for applying these tools is central to the success of new technologies. Fundamentally, promoting digital training in medical education may help equip HCPs with the necessary skills to implement and manage new technologies.

Although endorsement by clinicians is a key facilitator of patient acceptance of these tools, the role of the care team is also critical, especially after discharge. This is because they are best positioned to support problem-solving, considering their expertise in managing illness and excellent communication skills in establishing a therapeutic relationship with the patients (37). Furthermore, if the care team encourages patients to use mHealth-based systems but fails to monitor the data entered into the mHealth program actively, it would eventually lead to mHealth abandonment even if the user initially agrees to adopt the tool (38). This highlights the importance of integrating mHealth into the clinical workflow in the future so that the data generated by these tools can be used seamlessly in standard clinical practice.

Patients are often subject to the social influence such as the support from family members (29) and peers interaction (37), which is consistent with our findings. Interestingly, the availability of strong social support and regular caregiver may sometimes discourage adoption of the mHealth system, as they receive sufficient help from their caregivers and believe that mHealth is unnecessary (39). Additionally, because the family is a highly significant concept in Chinese culture, it is the duty of other family members to care for the ailing family members, support them, and encourage them to limit their activities. Moreover, despite acknowledging the advantages of exercise, they reject vigorous activity and classify all forms of exercise—except walking—as strenuous (13). Since the role of the family is important, educating family members, transforming their traditional attitudes and thoroughly understanding the importance of PA could help patients gain effective support. As for peer support, it is recommended that a face-to-face peer network be established in the hospital, which can be expanded as an online network after discharge and facilitated by a professional (40).

It is obvious that mHealth would reduce the reliance on HCPs and make interventions more accessible and cost-effective and was perceived by patients as a good complement to traditional face-to-face healthcare services (41, 42). However, the costs associated with using mHealth, such as the cost of internet access, remain a concern for patients. Despite the rapid increase in the use of smartphones in China, access to smartphones and the internet remains limited in some remote rural areas. Poor network services and wireless signal coverage are barriers to the mHealth program. The same problems exist in other countries; for example, in the UK, the cost of technology and poor access to free internet services prevent many people from participating in the mHealth program (43). Considering these problems, although China is strongly advocating internet-based healthcare, the government still needs to improve the infrastructure of network facilities. The manufacturers of the mHealth-based systems should also provide ongoing assistance services and usage guidelines to support patients in using mHealth applications. Moreover, encouraging reimbursement of costs generated by the mHealth system may help overcome the cost-related barriers.

To our knowledge, this is the first study to provide insights regarding the needs and preferences of HNC patients for developing mHealth-based PA interventions, thus filling the current knowledge gap. Rigorous and reliable qualitative methods were used to analyze the data, providing insights beyond those acquired quantitatively. However, this study has several limitations. First, the sample primarily comprised HNC patients from a hospital in northern China. The deep-rooted social and cultural issues highlighted in this population may not be prevalent in other contexts; therefore, case-to-case transferability may be limited in other contexts. Second, the results need to be strengthened further, as the patients were asked to provide their own needs and perceptions about future PA management applications rather than the developed application. Furthermore, since data collection was completed at one point in time, the needs and perceptions of HNC patients for future PA applications may change over time.

## 5 Conclusion

This study provides pragmatic ready-to-use recommendations to develop mHealth-based PA interventions for HNC patients. Future mHealth interventions should be tailored according to the needs of the patient, utilizing perceived facilitators and removing perceived barriers that enable HNC patients to engage in PA actively and improve their quality of life.

Further research is warranted to explore optimizing and tailoring interventions to address these considerations. Given the necessity of HCPs in intervention programs as perceived by patients, future studies should incorporate perspectives of the HCPs to customize mHealth PA interventions. Additionally, healthcare systems need to boost training and resource allocation to make the HCPs more proficient in technology and encourage more patients to participate in PA. Furthermore, strengthening the progression of information technology to reduce barriers perceived by patients and promote public health education is warranted.

## Data availability statement

The original contributions presented in the study are included in the article/Supplementary material, further inquiries can be directed to the corresponding authors.

## Author contributions

YN: Data curation, Formal analysis, Methodology, Writing – original draft, Writing – review & editing, Project administration, Supervision. ZD: Project administration, Resources, Writing – original draft, Formal analysis. ZJ: Formal analysis, Investigation, Writing – original draft. WZ: Formal analysis, Methodology, Writing – review & editing. YD: Methodology, Supervision, Writing – review & editing. QW: Formal analysis, Investigation, Methodology, Supervision, Writing – review & editing. RZ: Formal analysis, Methodology, Project administration, Supervision, Writing – review & editing. SH: Formal analysis, Methodology, Project administration, Resources, Supervision, Validation, Writing – review & editing.
